# The effects of slow wave sleep characteristics on semantic, episodic, and procedural memory in people with epilepsy

**DOI:** 10.3389/fphar.2024.1374760

**Published:** 2024-04-25

**Authors:** Yvonne Höller, Stefanía Eyjólfsdóttir, Frank Jasper Van Schalkwijk, Eugen Trinka

**Affiliations:** ^1^ Faculty of Psychology, University of Akureyri, Akureyri, Iceland; ^2^ Hertie-Institute for Clinical Brain Research, Center for Neurology, University Medical Center Tübingen, Tübingen, Germany; ^3^ Department of Neurology, Christian Doppler University Hospital, Member of the European Reference Network EpiCARE, Neuroscience Institute, Paracelsus Medical University and Centre for Cognitive Neuroscience Salzburg, Salzburg, Austria

**Keywords:** epilepsy, slow wave sleep, episodic memory, procedural memory, declarative memory, anti-seizure medication

## Abstract

Slow wave sleep (SWS) is highly relevant for verbal and non-verbal/spatial memory in healthy individuals, but also in people with epilepsy. However, contradictory findings exist regarding the effect of seizures on overnight memory retention, particularly relating to procedural and non-verbal memory, and thorough examination of episodic memory retention with ecologically valid tests is missing. This research explores the interaction of SWS duration with epilepsy-relevant factors, as well as the relation of spectral characteristics of SWS on overnight retention of procedural, verbal, and episodic memory. In an epilepsy monitoring unit, epilepsy patients (N = 40) underwent learning, immediate and 12 h delayed testing of memory retention for a fingertapping task (procedural memory), a word-pair task (verbal memory), and an innovative virtual reality task (episodic memory). We used multiple linear regression to examine the impact of SWS duration, spectral characteristics of SWS, seizure occurrence, medication, depression, seizure type, gender, and epilepsy duration on overnight memory retention. Results indicated that none of the candidate variables significantly predicted overnight changes for procedural memory performance. For verbal memory, the occurrence of tonic-clonic seizures negatively impacted memory retention and higher psychoactive medication load showed a tendency for lower verbal memory retention. Episodic memory was significantly impacted by epilepsy duration, displaying a potential nonlinear impact with a longer duration than 10 years negatively affecting memory performance. Higher drug load of anti-seizure medication was by tendency related to better overnight retention of episodic memory. Contrary to expectations longer SWS duration showed a trend towards decreased episodic memory performance. Analyses on associations between memory types and EEG band power during SWS revealed lower alpha-band power in the frontal right region as significant predictor for better episodic memory retention. In conclusion, this research reveals that memory modalities are not equally affected by important epilepsy factors such as duration of epilepsy and medication, as well as SWS spectral characteristics.

## 1 Introduction

For decades scientists documented the multifaceted effects of epilepsy on memory. Patients might suffer from impaired memory depending on the type of epilepsy (Elger et al., 2004; Muhlert et al., 2011), wherein temporal lobe epilepsy has repeatedly been shown to impair verbal memory performance (Helmstaedter, 2002). Notably, memory impairments may be caused by additional factors, such as the location of the seizure onset zone (Wilkinson et al., 2012), type and frequency of seizures (Wilkinson et al., 2012; Höller and Trinka, 2015), amount, type, and timing of interictal epileptic activity (Binnie, 2003; Holmes and Lenck-Santini, 2006; Fitzgerald et al., 2013), anti-seizure medication taken (Motamedi and Meador, 2004; Jokeit et al., 2005; Höller et al., 2020a), age of epilepsy onset (Thompson, P. J. and Corcoran, 1992), and comorbidities of psychiatric nature (Rayner and Tailby, 2017) such as depression (Brown et al., 1994) among others. Even a longitudinal decline of memory in a dementia-like manner was postulated, but only a high life-time number of tonic-clonic seizures exhibited such an effect (Dodrill, 1986).

Additionally, memory modalities may be differently affected by epilepsy-relevant factors. Specifically, verbal memory is frequently documented to be impaired in patients with temporal lobe epilepsy (Helmstaedter, 2002). The situation is, however, more complex than the old and frequently criticized “phrenological thinking” might suggest (Rayner and Tailby, 2017; Baxendale, 2018). This approach assumes that epileptic activity in one brain region affects memory that depends on the very same structure (Rayner and Tailby, 2017; Baxendale, 2018). Today, epilepsy is recognized as a network disease (Kramer and Cash, 2012), and the representation of memory is also understood to involve a complex network of cortical and subcortical components (McNaughton and Vann, 2022). Thus, multiple memory modalities can be affected by localized epileptic activity. For example, in addition to verbal memory, also episodic memory is affected in temporal lobe epilepsy (Helmstaedter, 2002).

To document the multifaceted problems of memory retention in people with epilepsy, a multitude of neuropsychological tests can be utilized (Wilson et al., 2015; Baxendale et al., 2019). However, these tests are usually completed in a single session with little time separating learning and delayed memory testing. It is increasingly recognized that people with epilepsy suffer from accelerated forgetting, impaired retention, or impaired consolidation (Kapur et al., 1997; Blake et al., 2000; Mameniskiene et al., 2006; Butler and Zeman, 2008; Fitzgerald et al., 2013; Cassel and Kopelman, 2019) Consequently, the validity of evaluating memory performance during a single session is put into question by evidence that self-rated memory performance correlates with recall after long delays (e.g., days), but not after a 30min delay (Fitzgerald et al., 2013). Accelerated long-term forgetting and remote memory impairment cannot be sufficiently determined by standard memory tests (Butler and Zeman, 2008). It is therefore important to investigate long-term retention in people with epilepsy, as well as the variables that undermine the patients’ capacity to consolidate and retain memory.

Sleep between learning and recall supports memory performance in healthy participants (Born et al., 2006), during which cardinal sleep oscillations facilitate reactivation of previously acquired information and memory consolidation processes (Ficca et al., 2000; Gais and Born, 2004). Sleep-dependent memory consolidation depends on the type of memory and the sleep architecture. Specifically, procedural memory (Schönauer et al., 2014), but also declarative and emotional memories were shown to benefit from sleep between learning and recall, with slow wave sleep (SWS) benefiting declarative memories more while rapid eye movement sleep enhances procedural and emotional memory (Diekelmann et al., 2009). Furthermore, sleep spindles were associated with better procedural memory consolidation (van Schalkwijk et al., 2020; Kumral et al., 2023; Boutin et al., 2024).

People with epilepsy are vulnerable to effects of impaired sleep-dependent memory consolidation (Latreille et al., 2022) because of the bi-directional relationship between epilepsy and sleep (Malow, 2007) and, more specifically, because of the negative effects of epilepsy on sleep architecture including fragmentation of slow wave sleep (López-Gomáriz et al., 2004). Among these effects, sleep-dependent interictal epileptiform events particularly during non-REM sleep (Urbain et al., 2011; Bjørnæs et al., 2013; Galer et al., 2015; Halász et al., 2019) and sleep-related seizures (Malow, 2007) partly explain hampered sleep-related memory consolidation in people with epilepsy. Additionally, anti-seizure drugs impact brain activity, e.g., by increasing or decreasing power in specific frequency bands (Höller et al., 2018; Höller and Nardone, 2021) and they can change sleep architecture by extending or shortening specific sleep stages (Carvalho et al., 2022). Thus, it is plausible that anti-seizure drugs also impact sleep-related consolidation of memory, but the exact nature of this relationship is poorly understood.

Among patients with left-lateralized temporal lobe epilepsy, verbal memory decays at an increased rate when a seizure occurs in the 24 h between learning and recall (Jokeit, H. et al., 2001). In N = 8 patients with transient epileptic amnesia (Atherton et al., 2014) and in a similarly small sample of N = 7 patients with temporal lobe epilepsy (Deak et al., 2011), it was found that verbal memory decayed more rapidly as compared to healthy control subjects during daytime retention, whereas no group differences were observed for overnight memory retention. In the whole group, verbal memory performance was significantly correlated with the percent of SWS in the entire night, but not in the subgroups, since they were too small (Deak et al., 2011). In another study, SWS duration in healthy controls was shown to be correlated with sleep-related benefits on overnight retention of verbal memory, whereas no correlation was observed in people with epilepsy (Atherton et al., 2016). The fact that multiple studies did not find nighttime sleep architecture to be related with memory retention in people with epilepsy (Fitzgerald et al., 2013) emphasizes the potential detrimental effects of epilepsy on memory retention.

Retention intervals separating learning and recollection are prone to disruptions of memory consolidation processes by epileptiform activity. In a 24 h retention interval, patients with temporal lobe epilepsy showed similar performance retention on a fingertapping task as compared to control subjects, but only one patient reported a seizure during this time period (Deak et al., 2011). Indeed, in another study only seizure-free retention periods were associated with improved performance, whereas no performance changes were observed when a seizure occurred during the retention period separating learning and delayed recall (van Schalkwijk et al., 2021). It is therefore important to control for the occurrence of seizures during retention intervals.

Finally, non-verbal memory was assessed in a card-position association task in older people with epilepsy, and it was found that 24 h retention was significantly predicted by the number of anti-seizure drugs, interictal epileptiform activity, as well as slow wave activity power during non-rapid-eye movement (non-REM) sleep (Sarkis et al., 2023). The importance of slow wave activity has also been documented based on intracranial hippocampal recordings, where delta activity (2–4 Hz) in the hippocampus during non-REM sleep was positively correlated with task performance at retest in a spatial memory task (Moroni et al., 2014). Another study investigated non-verbal declarative memory with pairs of pictures and found that overnight memory retention was positively correlated with the duration of SWS and showed that seizure occurrence impaired memory retention (Sarkis et al., 2016).

In summary, sleep plays an important role for the consolidation and retention of verbal, procedural, and non-verbal/spatial memory in people with epilepsy, wherein SWS in particular plays a prominent role for memory consolidation and retention. In contrast, seizures can lead to impaired overnight retention of procedural and non-verbal memory.

As a general limitation, the sizes of the patient samples in the studies examining overnight memory retention are all very small, limiting possibilities for statistical assessment of multiple moderating factors of overnight retention and therefore restricting overall conclusions (Latreille et al., 2022). It is still unclear whether SWS duration and spectral characteristics as well as other factors such as medication and seizures during the retention interval impact all memory modalities equally. To date, most studies have restricted themselves to studying the effects of epilepsy on memory retention for one or two memory modalities, and no ecologically valid tests have been employed for episodic memory. In the present study we addressed these issues by investigating a larger sample of people with epilepsy that underwent testing of verbal, procedural, and episodic memory, wherein an innovative and ecologically valid virtual-reality test was implemented to assess episodic memory. We examined whether verbal, procedural, and episodic memory are similarly affected by SWS duration and power spectra of SWS during the retention night, and crucially controlled for seizure occurrence during the retention night, medication (anti-seizure and psychoactive), depression, type of seizure, gender, and duration of epilepsy as the current age minus the age of onset of seizures.

## 2 Materials and methods

### 2.1 Ethics

The ethical commission of the Region of Salzburg, Austria approved the study protocol (approval nr. 415-E/1755/20–2016). The study was carried out in agreement with the Declaration of Helsinki. All patients signed written informed consent forms.

### 2.2 Setting and recruitment

The study took place at the Epilepsy Monitoring Unit (EMU) of the University Clinic for Neurology, Christian Doppler Medical Centre of the Paracelsus Medical University in Salzburg, Austria. The unit consists of four beds and a monitoring room, where trained staff monitor the patients’ electroencephalogram (EEG) and video throughout the examination. The daily rhythm in the epilepsy monitoring unit is structured by lights being turned on in the morning (06:30–07:00), breakfast (07:00–07:30), lunch (11.30), dinner (16:30), and lights being turned off in the evening (22:00–00:00). During the monitoring period it is common to taper the dosage of anti-seizure drugs and to expose patients to sleep deprivation in the third night to provoke seizure occurrence. Patients’ stay within the EMU typically lasted from Monday until Friday to obtain detailed recordings of suspected seizure events thus facilitating detailed diagnosis of their epilepsy syndrome and presurgical evaluation. Patients with a high likelihood of an epilepsy diagnosis were invited to participate in the study. Further inclusion criteria were age (between 18 and 75 years), fluency in the German language, adequate intellectual ability to give informed consent and perform the memory tasks, and no interfering neurological diseases of degenerative nature. Informed consent was obtained following admittance on Monday morning by medical staff, after which we recorded demographical information and patients were tested for depression with the German version of Beck’s Depression Inventory (Hautzinger et al., 2006). EEG was installed by medical technicians and continuously monitored and maintained for the full duration of stay within the EMU. A total of 106 patients were recruited between the second of February 2016 and the 11th of June 2018. Exclusion criteria were experience of status epilepticus during the stay, missing data, as well as unclear diagnosis.

### 2.3 Procedure

**FIGURE 1:**
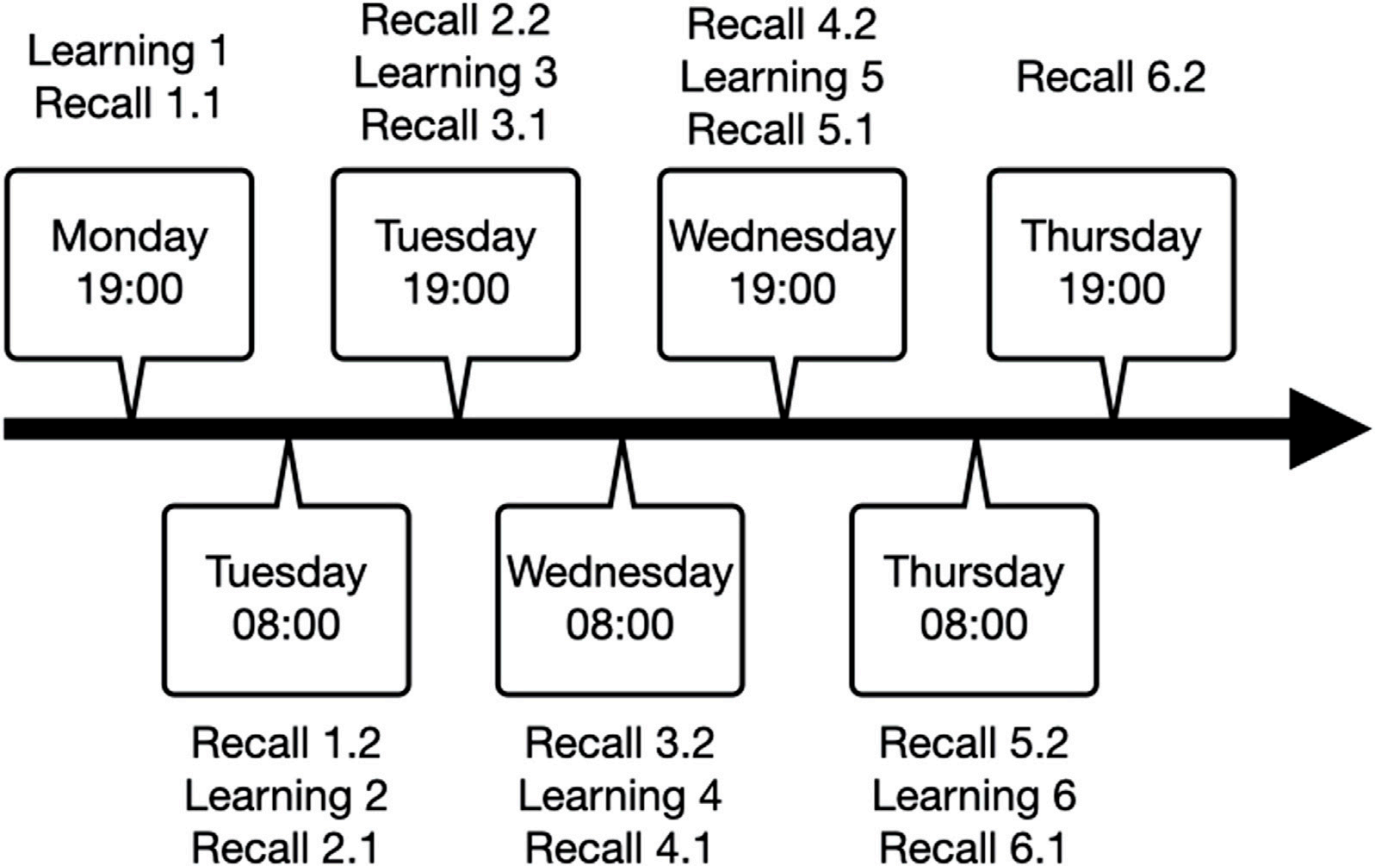
Repeated testing procedure with learning sessions, immediate recall (denoted as 1.1, 2.1, 3.1, etc.) and delayed recall (denoted as 1.2, 2.2, 3.2, etc.).

The study involved a total of seven sessions, with two testing sessions per day occurring at 08:00 and 19:00. The procedure that started on Monday evening and lasted until Thursday evening. Each session included three memory tasks that concerned verbal, procedural, or episodic memory. With exception of the first, each session started with recollection of task information learned during the previous session, followed by learning the new variations of the three memory tasks and immediate assessment of memory performance (Figure 1).

All testing was conducted at bedside using a laptop (17″ Lenovo) placed on the patient’s bedside table. The table was rotated such that the patient could look straight at the laptop screen and comfortably reach the keyboard.

### 2.4 Memory tasks

#### 2.4.1 Fingertapping task: Procedural memory

**FIGURE 2:**
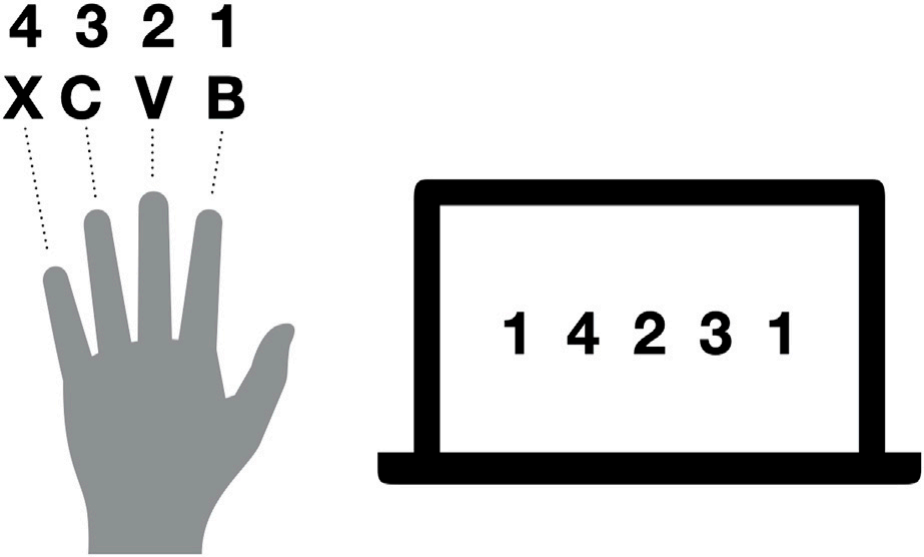
Exemplary sequence for fingertapping. Numbers indicate the allocation of fingers to numbers required to type the desired sequence, whereas letters indicate the keys on the keyboard used. Thus, for the illustrated sequence, the fingers were moved in the following order: ring finger, middle finger, index finger, little finger, ring finger.

The fingertapping task is described in full detail in a previous publication arising from the same project where the task was shown to reveal the effect of seizures on offline consolidation of procedural memory during day and night (van Schalkwijk et al., 2021). In short, patients were instructed to type a five-element sequence using four fingers of their non-dominant left hand as quickly and accurately as possible (see Figure 2). The learning session consisted of 12 trials of 30 s each with inter-trial breaks of 30 s. Recall sessions had the exact same procedure as learning sessions but consisted of four trials. Outcome parameters were the number of completed sequences per trial to indicate speed, and the percent of correctly typed sequences relative to the total keystrokes per trial as accuracy. As a performance measure that integrates speed and accuracy, we measured the number of correctly typed three-element sequences within a trial (triplets; e.g., for a sequence of 1-4-2-three to one, the triplets were1-4-2, 4-2-3, 2,3–1, 2-one to one, and 1-1-4). For each outcome measure, we averaged over the last three trials of the respective learning session to get immediate recall performance, and over the last three trials of the following recall session to measure delayed recall performance. Finally, we calculated performance changes from learning in the evening to recall in the morning as a ratio of the number of triplets during delayed recall divided by the number of triplets in the immediate recall.

#### 2.4.2 Word-pair task: verbal semantic memory

**FIGURE 3:**
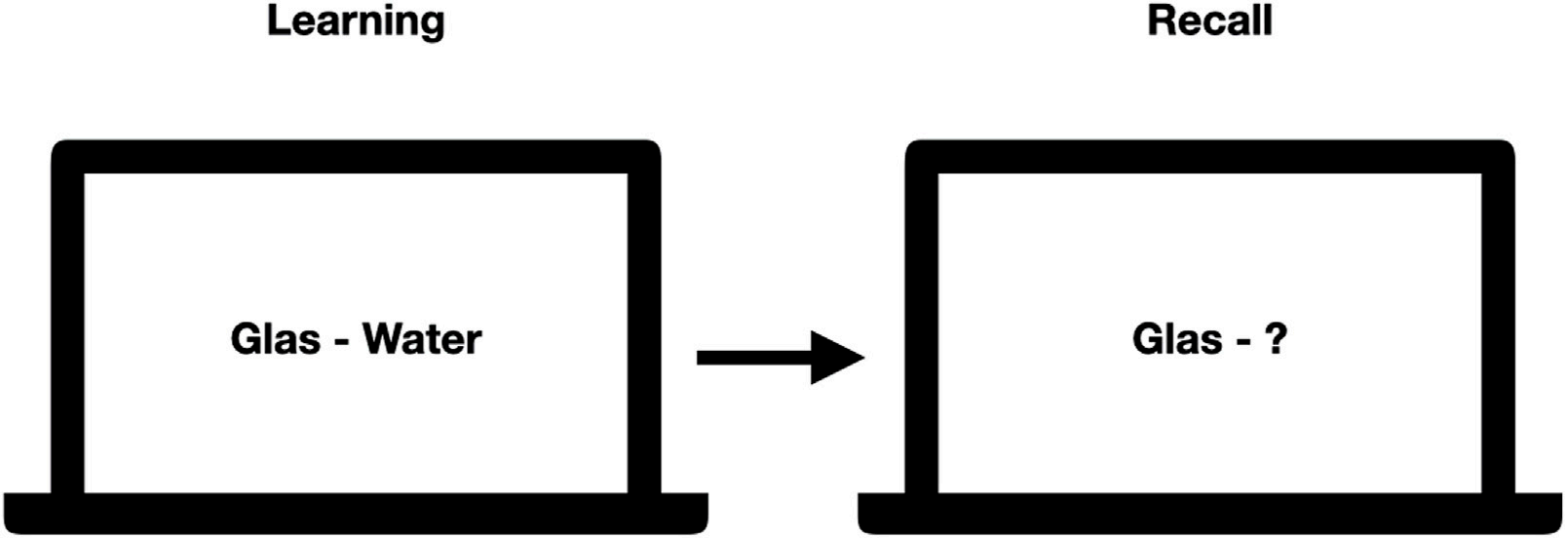
Exemplary learning and recall scene for the wordpair task. During learning, pairs of words were shown while during recall only the first word was shown, and the patients were asked to spell out the second word. Correctness of their answer was noted down by an experimenter.

For the word-pair task, six sets of 60 word-pairs were created based on the Berlin Affective Word List (Võ et al., 2009; Jacobs et al., 2015). From the list, we extracted nouns with an emotional valence mean between −1 and +1 (overall scale ranges from −3 to +3) and an arousal mean of 1.33–3.30 (overall scale ranges from one to 5), with two to three syllables. The six lists were balanced for these parameters. Each list consists of 60 word-pairs, of which 30 pairs are semantically related and 30 semantically unrelated. During learning, patients were presented with the session-specific word-pair list, wherein singular word-pairs were shown in a fixed order for a duration of 10 s per pair. To monitor attention and induce a similar learning strategy for all patients, they were instructed to think of a potential connection between the two words and rate the words to be connected or not. For example, word pairs were “glas - water” (related) or “floor - leaf” (unrelated). During recall, patients were shown only the first word of each word pair and were asked to report the second word or indicate that they had forgotten (see Figure 3).

A researcher with a copy of the respective word pair list noted whether the answer was correct. Trials were not time restricted and lasted until the participant gave an answer. Like in the fingertapping task, the outcome measure for the word-pair task was the ratio of the number of correctly remembered word-pairs in the morning during delayed recall divided by the number of correctly remembered word-pairs in the immediate recall session in the evening of the day before.

#### 2.4.3 Virtual reality task: episodic memory

**FIGURE 4:**
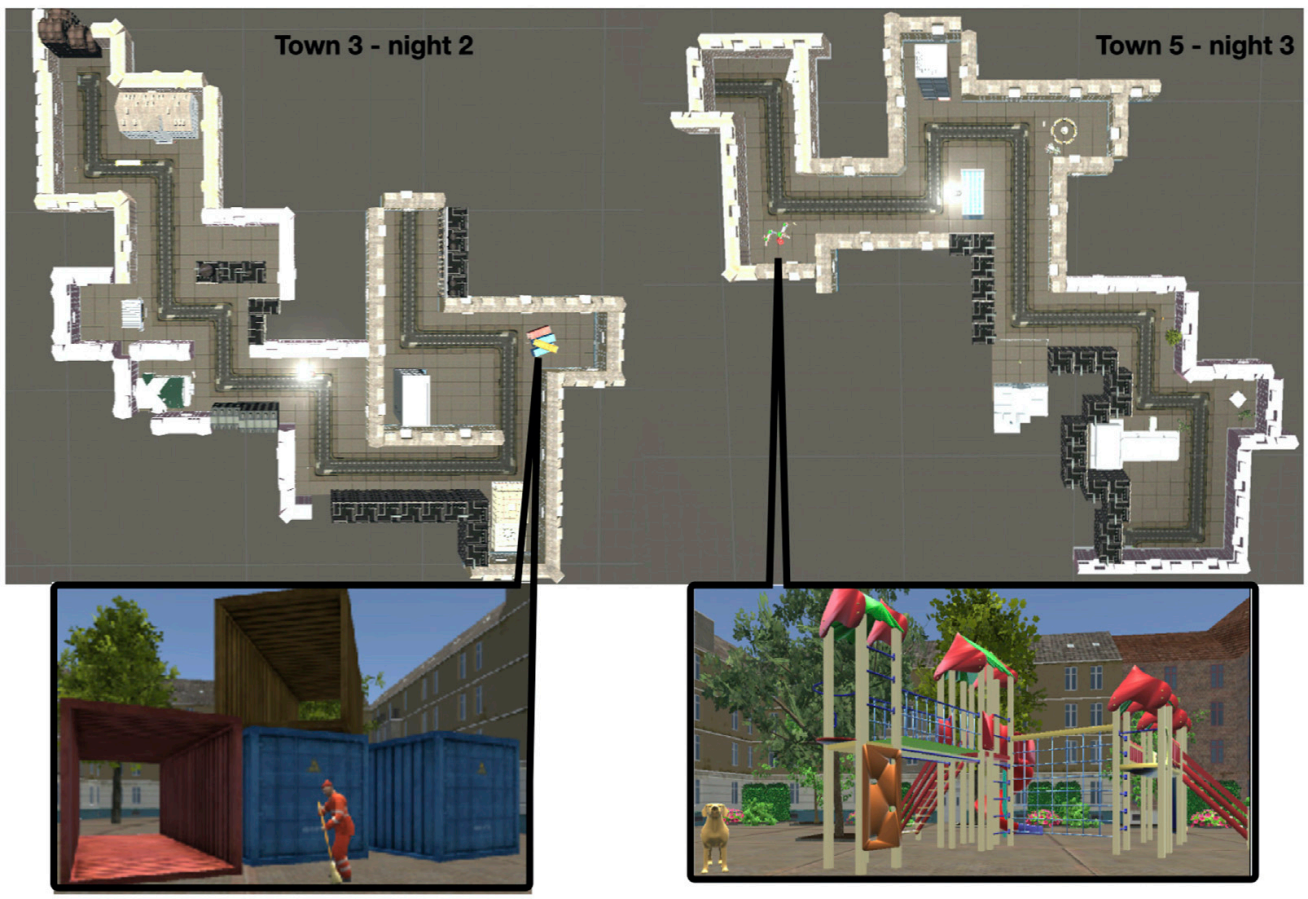
Outline of the paths for towns three and 5, studied in the evening of night two and 3, alongside with an example of one scene. The scene in town three shows containers with a man cleaning the street in front of them, the scene in town five shows a playground and a dog.

The virtual reality task was created for this project in UNITY (Unity Technologies ApS, Engine Version 5.3.5f1, Unity3d.com), including several packages (asset store products) to fill the towns with details. The task can be downloaded from the dataset referenced in the manuscript describing it in full detail (Höller et al., 2020b). The task was shown to respond to anti-seizure drug tapering in another publication arising from the same project (Höller et al., 2020a). In short, patients navigated through a virtual town on the computer screen, using the cursor keys on the keyboard to move forward, left, and right. The six towns had only one path that could be followed to explore the environment. Each town included 10 turns (left or right) which we call hereafter “scenes”. Each scene included at least two elements. The towns’ paths learned during night two and three are shown in Figure 4.

On the first day, patients completed a practice run in an empty town with no details to become familiar with the navigation. The practice lasted as long as needed to become familiar with the procedure. We examined episodic memory regarding remembered details, egocentric, and allocentric information. During immediate and delayed recall, patients were first asked to name all the elements they could remember, e.g., a car, a hospital, a park, a table. This number of elements constitutes what we denoted as “WHAT” outcome of memory in this task. Then, we requested additional descriptions of each element and counted the number of correctly remembered details. For example, if a patient indicated remembering a table, they would describe it by color, shape, texture, etc. The description of a hospital could include a red cross at the front of the building, number of floors, etc. The number of correctly remembered details constituted the “DETAILS” outcome of memory performance. Next, we asked for each remembered element when the patients would have seen it in the town, with the option to say rather at the beginning, middle, or end of the town. This ordering of elements constitutes the “WHEN” outcome. Based on the total number of scenes in each town, the first three scenes were considered to be at the beginning, the following four as in the middle, and the last three as the end of the town. Furthermore, patients were asked whether they turned left or right after the element. Whether the element was to their left or right constituted the “EGOCENTRIC WHERE” outcome. When patients remembered more than one detail in one scene, patients were asked about the allocation of the details relative to each other, constituting the “ALLOCENTRIC WHERE” outcome. To the numbers noted down for these memory outcome parameters WHAT”, “DETAILS”, “WHEN”, “EGOCENTRIC WHERE”, and “ALLOCENTRIC WHERE”, only correct responses were counted. The consensus-based evaluation strategy of the patients’ responses is described in further detail in our previous publication (Höller et al., 2020b).

To get one single outcome measure, we summed up the correctly remembered items across the subscales “WHAT”, “DETAILS”, “WHEN”, “EGOCENTRIC WHERE”, and “ALLOCENTRIC WHERE”. Like in the word-pair task, the outcome measure that was submitted to the statistical analysis was the ratio of the number of correctly remembered items in the morning during delayed recall divided by the number of correctly remembered items in the immediate recall session in the evening of the day before.

### 2.5 Drug load

We determined drug load at admission and on the day preceding the experimental night for psychoactive and anti-seizure drugs. For each drug we calculated the ratio between the prescribed dose and the defined daily dose. Then, we summed the loads over all drugs taken on that day, separately for psychoactive and anti-seizure drugs.

### 2.6 EEG recording

EEG was continuously recorded throughout the stay in the epilepsy monitoring unit from each patient. The recording was conducted with a routine clinical system (SystemPlus Evolution, SD LTM 46 Express Amplifier, Micromed S. p.A, Mogliano, Italy). There were 29 standard electrodes placed according to the 10–20 system, with the Fpz as ground and Oz as reference. At setup on the day of admission, impedances were ensured to be below 10 kΩ and daily monitoring of data and noise in the signal ensured that improvements were made to keep this quality standard. Data were digitized at 1,024 Hz and online filtered by 0.1 Hz high-pass and 50 Hz notch to remove line-noise. Additional electrodes were attached to measure differential electrocardiogram, electromyogram, and electrooculogram. The electromyogram at the chin and additional horizontal electrooculogram electrodes were mounted only in the evening and removed in the morning for the present study to facilitate sleep staging.

### 2.7 Data selection

For the present study, we discarded the first night because of the well-known bias resulting from habituation of the patient to the recording environment (Mayeli et al., 2022). We aimed at including the data from the second night, thus including learning session 3 with immediate recall 3.1 and delayed recall 3.2 (see Figure 1). However, if sleep scoring for the second night did not include SWS, the third night was considered for analyses, thus including learning session 5 with immediate recall five and delayed recall 5.1 (see Figure 1). The third night was also considered if patients underwent sleep deprivation on the second night. Patients who did not reach SWS or had a viable night without sleep deprivation were excluded from further data analysis.

### 2.8 Seizure identification

The EMU staff worked in shifts with medical technical nurses working during the day and trained students during the night. Seizure monitoring included the detection of seizures based on EEG and behavioral cues and an ictal testing that evaluated responsivity, sensation and perception, consciousness, memory, and basic cognitive skills as per a standardized testing protocol (Beniczky et al., 2016). The day-shift team routinely screened the 24 h EEG and video recordings offline and marked segments with clinical and subclinical seizures, as well as all events that were suspected to be seizures but turned out to be of a different nature. All marked segments were further examined by a neurologist to confirm the markings.

For the purpose of this study, a clinically trained EEG technician re-evaluated the occurrence of seizures in the EEG and video and classified the seizures into tonic-clonic seizures and other seizures. Time of seizure onset was compared to the retention interval (i.e., night two or 3) to rate if a seizure occurred between learning and recall.

### 2.9 Slow-wave sleep staging

Sleep staging was performed manually with the aim of identifying SWS, corresponding to stages 3–4 in the manual of Rechtschaffen and Kales (Rechtschaffen and Kales, 1968) and stage N3 in the new AASM (American Academy of Sleep Medicine) manual (Iber et al., 2007). To this end, night files were extracted from the clinical system and imported to the software Brain Vision Analyzer (Brain Products GmbH). SWS was identified by scrolling through the EEG in 20 s epochs as recommended for fragmented sleep (Schulz, 2008). A trained researcher set markers that defined the beginning and end of each segment initially, and all segments were then reviewed by an experienced EEG-researcher. The criterion for SWS was that slow waves of the frequency below 2 Hz with high amplitude, synchronized activity must occupy at least 50% of an epoch (Hori et al., 2001).

### 2.10 Quantitative EEG-analysis

We analyzed SWS segments quantitatively in two ways. First, we extracted total duration of SWS by summing up the duration of each marked segment in the examined night of a patient. Then, the SWS segments were divided into epochs of 4 s and each epoch underwent Fast Fourier Transform (resolution 0.25 Hz, non-complex output in microvoltage, half spectrum). The resulting power spectra were averaged across all available epochs and power density was extracted for frequency bands delta (0.5–4 Hz), theta (5–7 Hz), alpha (8–12 Hz), sigma (13–15 Hz), and beta (16–29 Hz) as unsigned, rectified values of mean activity for each band. Finally, we averaged across electrodes for areas frontal left (F3, F7, F11), frontal right (F4, F8, F12), central left (C3), central right (C4), temporal left (T7, T11), temporal right (T8, T12), parietal left (P3, P7, P11), parietal right (P4, P8, P12), occipital left (O1), and occipital right (O2).

### 2.11 Statistics

All statistics were carried out with R 2022–06–23 in R-Studio version 2022.02.3 + 492 (R Core Team, 2022). We reported median and range for ordinal variables and mean and SD for interval variables. To control for the possible effect of temporal lobe seizures, we reported descriptives for immediate and delayed recall as well as ratios for this sub-samples and compared these values between the two subgroups (temporal lobe seizures vs. other). For the three memory outcome parameters, the ratio between delayed recall in the morning and immediate recall in the evening for fingertapping triplets, word-pairs, and correctly remembered items in the episodic-memory task, we performed multiple linear regression with the R function “lm” to identify significant determinants of memory decline (or improvement) overnight. We conducted this analysis separately for patient characteristics and brain activity during SWS.

Candidate predictor variables among patient characteristics were the BDI score, seizure type (focal, generalized), drug load of anti-seizure drugs, drug load of psychoactive drugs, sex, total duration of deep sleep in minutes, whether a seizure occurred during the night, whether a tonic-clonic seizure occurred during the night, and duration of epilepsy as the current age minus the age of onset of seizures. Since this analysis involved three models for the three memory outcomes, the Bonferroni-corrected level of significance was .05/3, i.e., the critical alpha level was *p* < .017.

Second, we estimated multiple regression models with the same R function “lm” for each frequency band (delta, theta, alpha, sigma, and beta). Candidate predictor variables were EEG power measures in the regions frontal left, frontal right, temporal left, temporal right, parietal left, parietal right, central left, central right, occipital left, and occipital right. Again, we performed Bonferroni-correction, which was applied to the three memory outcomes and the five frequency bands, thus, the critical alpha level of .05/15 was *p* < .003.

For supporting the visual analysis of statistical trends, we created fitted lines to scatter plots for significant effects based on the R-function “predictdf” which uses a t-based approximation for outliers and the general-linear model (glm) for normal confidence intervals.

## 3 Results

### 3.1 Sample characteristics

From the total sample of 106 patients who were included into the study, the first patient was excluded because of a change of protocol, six patients were excluded because of incomplete EEG data, and five patients needed to be excluded because no SWS was detectable (neither in night two nor in night 3). Sub-analysis of this sample with respect to the impact of no SWS on memory was not conducted because of the diversity of sleep patterns in this subsample. Specifically, two of them did sleep but did not display any SWS, two of them had very little sleep overall, and one had no sleep at all. Another 10 patients were excluded because the extensive examination in the video-EEG monitoring unit did not lead to a conclusive diagnosis of epilepsy. A total of 11 patients were excluded because the diagnostic procedure led to the conclusion that they did not suffer from epilepsy. Another six patients were excluded because they underwent brain surgery in the past. The type of surgery and time since surgery was so heterogeneous in this sample that we refrained from a sub-analysis in this sample (4 years ago partial removal of left temporal lobe, 2 years ago unilateral removal of amygdala and part of hippocampus, 1 year ago craniotomy and resection of a cystis, 13 years ago removal of arterial-venous malformation temporo-median parahippocampal left (2x embolisation), 2 years ago removal of dyembryoplastic neuroepithelial tumour, 2 years ago removal of gangliogliom at right gyrus occipitotemoralis medialis). Then, since the fingertapping task was required to be done with the non-dominant left hand, another seven left-handed patients were excluded. Finally, 20 more patients were excluded because of missing data in the memory testing for the respective night. This was partly due to instances where the testing was skipped because the patient had a seizure right before the scheduled testing and no testing was possible, sometimes due to the medical routine in the monitoring unit not allowing the research team to conduct the testing within the set time-window, sometimes due to patients’ refusal to participate, and some data could not be collected because of technical problems with the equipment. Thus, a total of 66 patients were excluded from analyses.

In the remaining sample of 40 patients, there were two patients where we analyzed night three instead of night 2. According to the medical diagnoses in this sample, 31 patients were diagnosed with focal seizures (14 temporal lobe, 14 frontal lobe, 12 other localization; 14 with clear lateralization to the left hemisphere, 7 with a clear lateralization to the right hemisphere, and 5 with bilateral localization–the rest remained with unclear lateralization), and nine patients were diagnosed with generalized seizures. The average age of onset of seizures among all patients was 22.30 years (SD = 15.14). Among people with epilepsy, there were 23 with abnormal findings in magnetic resonance imaging. More details on the patients can be found in the Supplementary Material.

The final sample of 40 patients consisted of 22 women who were on average 32.27 years old (SD 12.67) and the 18 men who were on average 32.33 years old (SD 15.16). The median score on the Beck Depression Inventory was 7.5 (range 0–39).

### 3.2 Medication, seizures, and slow-wave sleep

Detailed information on specific medication (anti-seizure medication, psychoactive medication, and other) taken by each participant in this study on the day of admission is given in the Supplementary Excel File column K. On the day before the assessed night, there were eight patients who did not take any anti-seizure medication, 23 took one type, seven took two types and two took three. Among all patients on anti-seizure medication, the average drug load was 0.97 (SD 1.12). Regarding psychoactive medication, 36 took none, two patients took one type, and two took three types of psychoactive medication. Among all patients who took psychoactive medication, the average drug load was 1.51 (SD 1.24). In the assessed night, there were six patients with focal seizures who experienced at least one seizure, of which 2 were tonic-clonic seizures. No patient with generalized seizures experienced any seizures during the assessed nights. SWS in the examined night lasted on average for 78.43 min (SD 48.41).

### 3.3 Memory performance

Table 1 shows the average memory performance in the three tasks of the whole sample. On average, verbal memory for word-pairs and episodic memory for the virtual towns declined over night, while procedural memory for the fingertapping sequences increased. Table 2 shows the same values separately for patients with temporal lobe seizures vs. other seizures. These two groups differed only by their baseline performance in the fingertapping triplets, but not in the overnight retention (delayed performance differences are not significant after correcting for multiple comparisons).

**TABLE 1:** Average memory performance in the three tasks for procedual memory (fingertapping), verbal memory (word-pairs), and episodic memory (virtual reality town).

Measure	Mean	SD	Median	Range
FT triplets immediate	72.03	36.30	75	2.67–153.33
FT triplets delayed	74.96	38.27	78	1–154
FT triplets ratio*	106.57	33.64	102.25	5.77–222.22
Word-pairs immediate	21.73	11.35	19	6–51
Word-pairs delayed	18.90	11.21	17	3–46
Word-pairs ratio*	85.12	19.03	92.58	30–111.11
Episodic immediate	41.45	14.92	42.5	3–74
Episodic delayed	34.63	17.39	35	8–82
Episodic ratio*	86.67	41.47	88.76	18.75–300

FT: fingertapping; *ratio is represented as %: = delayed/immediate *100.

**TABLE 2:** Average memory performance in the three tasks for procedual memory (fingertapping), verbal memory (word-pairs), and episodic memory (virtual reality town) separately for people with temporal lobe seizures vs. other seizures. Descriptives are given as median/range and test values as Mann-whitney U for ordinal variables (immediate, delayed) and as mean/SD and t-values for interval variables (ratios and FT triplets).

Measure	Temporal lobe seizures	Other seizures	Test value	*p*-value
FT triplets immediate	90.21 (33.09)	62.24 (34.66)	−2.51	.018
FT triplets delayed	91.57 (36.48)	66.01 (36.82)	−2.11	.045
FT triplets ratio*	1.00 (0.12)	1.10 (0.41)	1.11	.274
Word-pairs immediate	20 (10–34)	16 (6–51)	178.5	.932
Word-pairs delayed	17.5 (3–33)	14 (3–46)	192.5	.777
Word-pairs ratio	0.81 (0.24)	0.87 (0.16)	0.79	.441
Episodic immediate	45.5 (25–66)	40 (3–74)	161.5	.570
Episodic delayed	29.5 (8–82)	39 (15–53)	162.5	.590
Episodic ratio*	0.84 (0.21)	0.88 (0.49)	0.36	.718

FT: fingertapping; *ratio is represented as %: = delayed/immediate *100.

### 3.4 Regression models for memory performance

**FIGURE 5:**
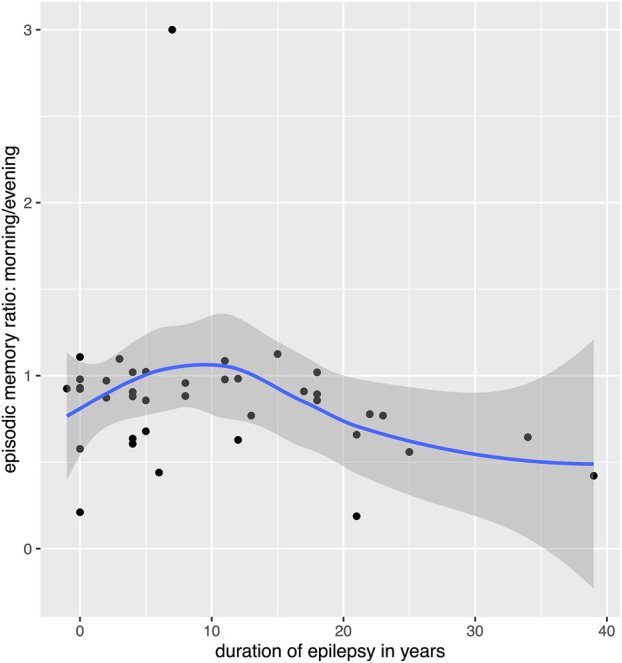
Relation between overnight memory retention for episodic memory and duration of epilepsy. Please note that the scatterplot puts points that are horizontally or vertically at the exact same number (e.g., 0) slightly displaced so the individual data points can be seen, this causes a point to appear before 0 years on the *x*-axis.

Table 3 shows the results of the multiple linear regression models estimates for the three types of memory outcome. None of the candidate variables significantly predicted overnight performance changes for procedural memory. For verbal memory, the only significant predictor was the occurrence of a tonic-clonic seizure, which significantly worsened the ratio of recalled words. However, only two patients experienced tonic-clonic seizures, and this result must therefore be interpreted with caution. We also observed a tendency for significantly lower verbal memory retention with higher load of psychoactive medication, which was significant before but not after correcting for multiple comparisons and which needs to be considered with caution because of the small number of patients taking psychoactive medication. For episodic memory, the only significant effect was the duration of epilepsy, which negatively impacted retention. However, an additional analysis revealed that this effect might not be perfectly linear but worsening of memory retention appears after epilepsy duration of 10 years (see Figure 5).

**TABLE 3:** Regression results for overnight retention of procedural memory as fingertapping triplets, verbal memory as word pairs, and episodic memory according to the virtual-reality test, predicted by the variables depression (BDI), anti-seizure medication drug load, psychoactive medication drug load, sex, SWS duration in minutes, seizure occurrence during retention night, tonic clonic seizure occurrence during retention night, and duration of epilepsy as age at testing minus age of onset.

	Regression coefficient	Std. Error	t-value	*p*-value
FT triplets ratio^a^				
BDI	−0.003	0.007	−0.369	.715
Anti-seizure medication drug load	0.012	0.059	0.210	.835
Psychoactive medication drug load	.004	0.110	0.038	.970
sex	−0.155	0.127	−1.219	.232
SWS duration	<0.001	<.001	0.800	.430
Seizure in night	−0.060	0.205	−0.294	.771
Tonic clonic seizure in night	−0.087	0.361	−0.242	.810
Duration of epilepsy	−0.007	0.007	−1.006	.322
Word pairs ratio^b^				
BDI	−0.003	0.003	−0.786	.438
Anti-seizure medication drug load	−0.014	0.026	−0.545	.590
Psychoactive medication drug load	−0.111	0.050	−2.241	.032
sex	−0.056	0.057	−0.989	.330
SWS duration	<0.001	<.001	0.823	.417
Seizure in night	0.139	0.092	1.515	.140
**Tonic clonic seizure in night**	**−0.534**	**0.162**	**−3.292**	**.002**
Duration of epilepsy	<.001	0.003	−0.021	.983
Episodic memory ratio^c^				
BDI	−0.008	0.008	−1.087	.286
Anti-seizure medication drug load	0.121	0.062	1.937	.062
Psychoactive medication drug load	−0.004	0.117	−0.039	.969
sex	−0.191	0.135	−1.416	.167
SWS duration	<.001	<.001	−2.118	.042
Seizure in night	−0.253	0.217	−1.163	.254
Tonic clonic seizure in night	0.466	0.383	1.216	.233
**Duration of epilepsy**	**−0.021**	**0.007**	**−2.790**	**.009**

FT: fingertapping; BDI: beck depression inventory score; SWS: SWS.

^a^ Triplets ratio model had a residual standard error: 0.3601 on 31 degrees of freedom.

^b^ Word-pair ratio model had a residual standard error: 0.1616 on 31 degrees of freedom.

^c^ Episodic ratio model had a residual standard error: 0.3816 on 31 degrees of freedom.

Bold font indicates significant results after correction for multiple comparisons.

A trend could be observed for total duration of deep sleep and episodic memory, which was significant before but not after correcting for multiple comparisons. Contrary to our hypothesis, longer SWS duration was associated with a lower ratio of recalled episodic memory in the morning. Another trend was observed for anti-seizure medication, which tended to have a beneficial effect on episodic memory.

Table 4 shows the results of the multiple linear regression for EEG band power during SWS with a *p*-value <.100.

**TABLE 4:** Regression results for overnight retention of procedural memory as fingertapping triplets, verbal memory as word pairs, and episodic memory according to the virtual-reality test, predicted by EEG power spectra. Only results with *p* < 100 are shown, see Supplementary Material for full list of coefficients and model residuals.

	Regression coefficient	Std. Error	t-value	*p*-value
FT triplets ratio				
Parietal left theta	−3.513	1.711	−2.053	.049
Central left theta	2.765	1.127	2.453	.020
Central right theta	−2.178	1.104	−1.974	.058
Central left alpha	4.081	1.418	2.877	.007
Central right alpha	−4.359	1.785	−2.442	.021
Word pairs ratio				
Central left delta	0.257	0.137	1.881	.070
Central right delta	−0.300	0.174	−1.725	.095
Central right theta	−1.32	0.748	−1.766	.088
Temporal right alpha	−2.639	1.512	−1.745	.092
Central right alpha	−2.424	1.092	−2.218	.035
Episodic memory ratio				
Temporal left delta	0.783	0.330	2.373	.025
Frontal left alpha	5.517	2.617	2.108	.044
**Frontal right alpha**	**−9.900**	**2.941**	**−3.367**	**.002**
Temporal right alpha	5.763	2.568	2.244	.033
Central left alpha	2.920	1.475	1.980	.057
Frontal left sigma	7.459	3.749	1.990	.056
Frontal right sigma	−10.906	4.528	−2.408	.023
Frontal left beta	21.621	9.152	2.362	.025
Frontal right beta	−26.471	11.599	−2.282	.030

FT: fingertapping; Bold font indicates significant results after correction for multiple comparisons.

The only one parameter turning out to be a significant predictor after correcting for multiple comparisons for the retention rate of episodic memory was lower SWS alpha-band power in the frontal right region (see Table 4).

Other trends observed in this memory domain were such that higher power over frontal left areas - but lower power over the frontal right areas - in the sigma and beta band were associated with better overnight retention (see Table 4). Additionally, higher delta power in the temporal left area, and higher alpha power in the temporal right and central left area, were associated with better overnight retention.

For the other memory types, only trends could be observed (see Table 4). For procedural memory, a trend was observed over central regions in the theta and alpha band, with higher power over the left central area and lower power over the right central area being associated with better over-night retention. Another trend was observed for lower parietal left theta power during SWS being associated with higher over-night retention.

For verbal memory, trends were small, with the only one with *p* < .05 (i.e., significant before but not after correction for multiple comparisons) such that better overnight-retention was associated with lower central right alpha band power during SWS (see Table 4).

## 4 Discussion

The present study sought to evaluate how overnight memory retention for procedural, verbal, and episodic memory modalities were associated with SWS duration, medication, seizure occurrence, duration of epilepsy, and EEG spectral properties of SWS in people with epilepsy.

### 4.1 Duration of epilepsy

Concerning episodic memory, the duration of epilepsy surfaced as a notable factor negatively impacting memory retention. Interestingly, an additional analysis hinted at a nonlinear relationship, suggesting that memory retention decline might manifest notably after an epilepsy duration of 10 years. The identification of a possible nonlinear relationship, with significant memory decline observed after a duration of 10 years, corroborate with prior literature indicating no negative impact of epilepsy duration on non-verbal memory retention (Jokeit et al., 2005; Fitzgerald et al., 2013). Nevertheless, our results are relevant given that an early age of seizure onset has been associated with a high risk for cognitive impairment (Rayner et al., 2016). Furthermore, the duration of epilepsy can indirectly impact memory performance and is affected by seizure frequency (Bergin et al., 2000; Rayner et al., 2016). However, these longitudinal studies do not explain impaired overnight retention as found in our study. We suggest that prolonged epilepsy duration manifests itself in consolidated epilepsy networks that could lead to permanent alterations in sleep architecture, including SWS, and subsequently affect overnight episodic memory retention.

### 4.2 Seizures

Regarding verbal memory, the occurrence of tonic-clonic seizures emerged as a significant predictor, significantly worsening the ratio of recalled words. However, only two patients experienced tonic-clonic seizures, limiting the generalizability of this result. It is important to note that analysis of any type of seizures did not emerge with a comparable effect. The observed impact of tonic-clonic seizures on verbal memory aligns with prior research suggesting that seizure activity can detrimentally affect memory performance. However, the limited occurrences of tonic-clonic seizures in our study cohort underscore the need for caution in generalizing this finding.

Early research on the effect of seizures found no effect of seizures occurring after learning on subsequent recall performance (Bergin et al., 1995). Indeed, the type of seizure is important in this respect as tonic-clonic seizures or generalized seizures might disrupt sleep more significantly than focal seizures, and therefore decrease quality of sleep and subsequent memory consolidation processes (Thompson et al., 2005). Furthermore, for verbal memory it was reported that seizures during a 24 h retention interval impair memory retention for left-sided but not right-sided temporal lobe-epilepsy (Jokeit et al., 2001). Thus, the type of seizures and nature of the epileptic disorder moderates the interaction between seizures and memory retention.

### 4.3 Medication

Trends were observed linking anti-seizure medication to better overnight retention of episodic memory. While high doses of anti-seizure drugs (Jokeit et al., 2005) and a large number of anti-seizure drugs (Lutz and Helmstaedter, 2005) negatively affect memory, positive effects of anti-seizure medication have been documented (Czubak et al., 2010), up to the extent where forgotten material was recovered thanks to initiation of pharmacological treatment (Midorikawa and Kawamura, 2007). Some anti-seizure medications can also affect sleep architecture in various ways (Liguori et al., 2021). Specifically, lamotrigine (N = 4 in our sample) and oxcarbazepine (N = 4 in our sample) bear the risk of worsening sleep, levetiracetam (N = 25 in our sample) was reported to have no effect on sleep, while lacosamide (N = 3 in our sample) and perampanel (N = 2) might even improve sleep (see Supplementary Table S1 for full details on medications). Changes in SWS duration (increase or decrease) or sleep quality due to medication side effects might influence memory consolidation in people with epilepsy, depending on the type of medication (Feld et al., 2013). According to our data, the positive trend observed for anti-seizure medication improving episodic memory retention was accompanied by a trend of decreased memory performance with longer SWS duration, which suggests that the potential impact of medication needs to be evaluated alongside with additional, possibly independent effects of SWS duration.

Additionally, a trending association between higher psychoactive medication load and lower verbal memory retention was observed. Although not statistically significant after multiple comparison corrections and affected by a small number of patients under this medication, this trend implies a potential influence of psychoactive medication on verbal memory but requires further investigation with a larger sample size for validation. Previous studies exploring the relationship between psychoactive medication and memory retention revealed differential effects of improvement or impairment of memory depending on the type of medication (Doss et al., 2023). Our findings are based on an overall drug load and are therefore mixing medications with diverse types of action together, limiting the overall conclusion on a potential negative association between higher psychoactive medication load and verbal memory. It is interesting to note that the trend for worse memory retention with higher psychoactive drug load emerged in the absence of effects from depressive symptoms, as the first line of interpretation would be that individuals taking antidepressive medication (N = 3 in our sample, two sertraline and one escitalopram) are most likely depressed, and depression leads to worse memory retention (Dillon and Pizzagalli, 2018). Therefore, it should be considered that memory impairment is more likely to be explained by a side effect of SSRIs, especially escitalopram (Anagha et al., 2021). However, SSRIs taken by the patients in our sample are rather associated with a positive impact on SWS Also, a negative memory impact is commonly reported after administration of sedative drugs such as benzodiazepines (Doss et al., 2023), which were taken by N = 2 patients in our patient sample (N = 1 Triazolam, N = 1 Clobazam). Benzodiazepines suppress SWS (de Mendonça et al., 2023), which impacts memory negatively. Another drug that could play a role in this respect is Quetiapine, as it was reported to negatively impact memory (Mori et al., 2004) but it was also taken by only N = 2 patients in our sample. The effects of Quetiapine on sleep are controversially discussed (Lin et al., 2023) and there is indication that it negatively impacts SWS (Monti et al., 2017). In conclusion, the sample size of patients taking psychoactive drugs was too small to conduct further sub-analyses by type of medication. Nevertheless, our results suggest that it might be important to control for concurrent effects of psychoactive drugs when examining memory retention in people with epilepsy.

### 4.4 SWS duration

In this study, trends were observed linking a longer duration of SWS to worsening of retention of episodic memory, but no effect of SWS duration was observed for procedural memory and verbal memory. This finding in people with epilepsy contradicts previous research in healthy individuals, wherein SWS is recognized to play a crucial role in memory consolidation (Rasch and Born, 2013). Previous work in healthy populations documented the significant impact of longer duration of SWS on better overnight memory retention (Diekelmann et al., 2012). This is especially true for consolidation of declarative memory, which includes episodic and semantic memory (Diekelmann et al., 2009). Episodic memory is more dependent on SWS (Berres and Erdfelder, 2021) than procedural memory (Ackermann and Rasch, 2014). For non-verbal declarative memory, SWS duration was reported to be clearly related to better over-night retention in people with epilepsy (Sarkis et al., 2016). Thus, while our results regarding procedural memory are in line with previous research in healthy controls, the lack of a link between SWS duration and verbal declarative memory as well as a trend for a negative association between SWS duration and episodic memory retention is unexpected.

People with epilepsy often experience disruptions in their sleep architecture (Bernard et al., 2023). However, previous research found no correlation between nighttime sleep architecture or interruptions of sleep and a 24 h delayed recall of verbal and non-verbal memory in people with epilepsy (Fitzgerald et al., 2013). This is in line with a study testing verbal memory with a word-pair task in healthy controls, showing that interrupting SWS did not affect overnight memory retention (Genzel et al., 2009). In contrast, among individuals with subjective cognitive complaints, which is a risk factor for experiencing further decline to mild cognitive impairment, fragmented SWS is related to poor verbal memory retention (Manousakis et al., 2019). When assessing verbal memory on the day after the examined night, the percent of time spent in SWS correlated positively with performance (van Schalkwijk et al., 2018).

While the result of the present analysis is only a trend, we speculate that longer SWS duration might be a result of the fragmented episodes of deep sleep. In order to respond to the need for SWS, people with epilepsy might present with more attempts to reach satisfying SWS, but because these SWS episodes are often interrupted by epileptiform activity, this fragmentation leads to an overall longer duration of SWS, which is not beneficial for memory consolidation. This speculation is supported by previous findings relating frequent interictal spikes during non-REM sleep to a reduced homeostatic decrease in the slope of sleep slow waves during the night and to reduced daytime learning (Boly et al., 2017). We suggest that future studies investigate the fragmentation of SWS in people with epilepsy in more detail, elucidating the potential interaction between SWS duration, fragmentation, and memory effects.

### 4.5 Power spectra of SWS

While our data suggested trends for relationships between neural oscillations during SWS and overnight memory retention, these associations did not consistently reach statistical significance. Only the retention rate of episodic memory was significantly higher when alpha-band power in the frontal right region was lower during slow-wave sleep. Interestingly, this was accompanied by a trend of increased left-frontal alpha-band power being associated with higher memory retention. Episodic memory consolidation during sleep is theorized to be reflected by a specific thalamic-hippocampal-neocortical interplay that depends on exact timing of slow and fast oscillations (Inostroza and Born, 2013; Klinzing et al., 2019). Alpha-band power has been linked previously to neuronal memory reprocessing during SWS (Yordanova et al., 2012). Also in this previous study, there was a stronger effect over the right hemisphere, but with increased alpha power being correlated with better knowledge transformation. Since the alpha band with 8–12 Hz overlaps partly with slow sleep spindles which also peak frontally (Anderer et al., 2001), it is plausible that the effect found in the present study is explained by sleep spindles, rather than EEG background activity. Slow spindles at 8–12 Hz occur during SWS and it was documented that increased slow spindle activity is related to better declarative memory performance (Marshall et al., 2006). The relevance of alpha activity during sleep was brought into context with the use- or experience-dependent theory of sleep function (Yordanova et al., 2012). According to this theory, homeostatic responses during sleep are related to learning before sleep (Borbély, 1982; Sejnowski and Destexhe, 2000), where the alpha band is associated with motor learning (Salih et al., 2009). A left-over right dominance was hypothesized to reflect the right-hand use in the experimental task (Yordanova et al., 2012), which is in line with the situation presented in our episodic learning task. While we did not intentionally include procedural learning, it might be that the keyboard-dependent navigation in the virtual town with the right hand is reflected in the alpha-dependent consolidation.

This observation is partly in contradiction with an additional observation derived from the fingertapping task. This task aimed at examination of procedural memory and was conducted with the non-dominant left hand. Also in this task was a trend of central theta and alpha activity being linked to better overnight consolidation, most of which significant before, but not after correction for multiple comparisons. Here, the strongest association was represented by increased central left alpha activity being linked to better memory consolidation. Interestingly, the tendency was such that increased left-hemispheric but lower right-hemispheric activity was associated with better overnight retention. We speculate that this finding reflects consolidation and replay of procedural memory over the motor-cortex–however, the hemispheric pattern is similar to the episodic memory task, where the other hand was used. This implies that the trending left-right asymmetry is not specific for a memory modality. While our results must be interpreted with caution given the non-significance after correction for multiple comparisons, we could speculate that the hemispheric pattern might reflect overall memory processing during SWS which is not necessarily linked to the hand used in the task.

For verbal memory, the trends were all very weak but pointed to lower central right activity in the delta, theta and alpha band being associated with better memory retention, which is in contrast with earlier research pointing towards higher activity in a broad frequency range being associated with better verbal memory retention (Holz et al., 2012). However, only left-hemispheric activity was investigated in this study. Another recent study suggests that the difficulty of the task matters (Qian et al., 2022). This recent finding related performance in easy declarative and procedural memory tasks with lower relative power of alpha or theta, but for a difficult version higher relative alpha band activity was associated with better performance.

### 4.6 Limitations

In view of the lack of associations with the parameters selected in this study it is possible that the most important factor for impaired learning among people with epilepsy was not included in our study. Specifically, in our study, we did not investigate interictal epileptiform discharges, which have been shown to play an important role in accelerated long-term forgetting of verbal information for intervals between 30min and 4 days (Fitzgerald et al., 2013). Future studies should analyze the contribution of interictal epileptiform discharges in relation to the spectral content of SWS in more homogeneous patient groups.

We were not able to control for all factors that might be considered a potential confounder. It is possible that the baseline difference between people with temporal lobe seizures vs. other seizures in the fingertapping task affected the overall result, although there was no such difference in the overnight retention ratio between these subgroups. Another limitation is that results could be skewed if the baseline intelligence was interacting with any of the parameters of interest. Unfortunately, we did not have test for the IQ, nor was this information obtained during the standard testing procedure. Furthermore, we did not distinguish patients with temporal lobe seizures from other subgroups, such as frontal lobe seizures, because of the small sample size. Despite mostly verbal memory impairment is best documented for temporal lobe seizures, memory impairment has also been observed in patients with frontal lobe seizures (Centeno et al., 2010). However, verbal memory retention is differentially affected by seizures in temporal lobe epilepsy depending on the lateralization of the epileptic focus (Jokeit, H. et al., 2001). In sum, our results need confirmation in larger and more homogeneous patient groups.

Concerning procedural memory, the study revealed that none of the candidate variables significantly predicted changes in procedural memory performance overnight. However, it is essential to consider the possibility of other unexamined variables or interactions that might contribute to procedural memory processes, such as localization of seizures and interictal epileptiform activity.

In this study we identified slow-wave sleep segments manually and did not investigate other sleep stages. Therefore, no further information on sleep architecture is available in the present work. In the future, automated sleep scoring will most likely replace the time-consuming task of sleep staging (Fiorillo et al., 2019). However, automated sleep scoring might not be accurate in epilepsy, as epileptiform activity can represent confounders for algorithms that have been developed for sleep staging in healthy individuals (Amin et al., 2023). The present approach is therefore preferable given the current state of the art of sleep staging.

It would have been interesting to compare the group without an epilepsy diagnosis to patients regarding their memory performance. However, after excluding participants in this sub-group with missing data in the memory tests, only one participant was left, which did not allow for such an analysis.

Although the current sample is large given the clinical limitations, further research with larger cohorts, more homogeneous subgroups and longitudinal designs is warranted to validate these findings and unravel the underlying mechanisms driving memory retention in people with epilepsy. We could not investigate more subtypes of seizures and medications because of this limitation, which represents an important limitation in the generalizability of the results.

### 4.7 Conclusion

The presented findings reveal relevance of epilepsy duration, medication, and SWS spectral contents for specific forms of memory in people with epilepsy. While the relation between tonic-clonic seizures and lower verbal memory retention was expected, a trending relation between extended duration of SWS and a decrease in episodic memory retention was surprising but is possibly due to fragmentation of SWS. Furthermore, the trends observed for positive effects of anti-seizure medication on episodic memory retention but negative effects of psychoactive medication on verbal memory retention warrant further investigation, given their potential relevance for neuropsychological testing of epilepsy-related memory deficits. Finally, we deem the negative association between frontal right SWS alpha activity and episodic memory interesting. This frequency band is possibly reflecting the activity of slow sleep spindles, and further trends in our data revealed an interesting asymmetrical pattern of higher left-sided and lower right sided frontal activity in this frequency band being linked with better memory performance. Since motor consolidation theories do not fully explain the pattern we found, we suggest investigating further the hemispheric distribution of slow sleep spindles during SWS and their relation to memory performance in the procedural and episodic domain.

## Data Availability

The raw data supporting the conclusion of this article will be made available by the authors, without undue reservation.
